# TLR4/ROS/miRNA-21 pathway underlies lipopolysaccharide instructed primary tumor outgrowth in lung cancer patients

**DOI:** 10.18632/oncotarget.9902

**Published:** 2016-06-07

**Authors:** Xianqi Zhang, Chunhong Wang, Shan Shan, Xiyu Liu, Zhongmin Jiang, Tao Ren

**Affiliations:** ^1^ Department of Thoracic Surgery, Qianfoshan Hospital, Shandong University, Shandong 250014, China; ^2^ Department of Respiratory Medicine, East Hospital, Tongji University School of Medicine, Shanghai 200120, China; ^3^ Department of Thoracic Surgery, Affiliated Hospital of Guilin Medical University, Guilin 541001, China

**Keywords:** lung cancer, LPS, TLR4, miR-21, ROS

## Abstract

Activation of Toll-like receptor 4 (TLR4) signaling in human lung cancer with lipopolysaccharide (LPS) enhances tumor progression. However, whether primary human lung cancer outgrowth could respond to LPS and underlying mechanisms are unclear. Here we determined that TLR4 activation with LPS promoted outgrowth of primary human lung cancer cells *in vitro* and *in vivo*. Mechanistically, LPS stimulation increased expression levels of microRNA-21 (miR-21) in primary human lung cancer cells. Inhibition of miR-21 blocked the enhanced lung cancer growth induced by LPS *in vitro* and *in vivo*. Up-regulation of miR-21 promoted outgrowth of primary human lung cancer. Down-regulation of miR-21 limited primary human lung cancer outgrowth. Further, TLR4 activation in primary human lung cancer cells increased their ROS levels. Scavenge of ROS abrogated the up-regulation of miR-21 in primary human lung cancer cells and attenuated LPS-induced outgrowth. For *in vivo* relevance, expression of TLR4 was correlated with miR-21 expression and ROS production in freshly isolated, untreated primary human lung cancer cells. These findings demonstrate an essential role of TLR4/ROS/miR-21 pathway in LPS-induced outgrowth of primary human lung cancer. Our study connected a framework of innate signaling, oxidative stress and microRNA in tumor immunity and provided clues for developing new therapeutics for lung cancer.

## INTRODUCTION

Lung cancer is the leading cause of cancer deaths worldwide [1]. Currently, the outcome of lung cancer patients still remains poor, thus, exploration of crucial effectors involved in cancer progression was urgently needed [2, 3]. Recently, accumulating studies reported that pulmonary infection facilitated lung cancer progression [4]. Lipopolysaccharide (LPS), a major component in the outer membrane of Gram-negative bacteria, directly promotes the tumor progression [5–7]. Expression of Toll-like receptor 4 (TLR4), which recognizes LPS from Gram-negative bacteria, was increased and reflected disease progress of lung cancer patients [8, 9]. These findings regarded TLR4 activation by LPS as an important stimulus of lung cancer progression. However, whether primary human lung cancer outgrowth respond to LPS and underlying mechanisms remain unclear.

MicroRNAs (miRs), which are small non-coding RNA molecules capable of regulating gene expression at posttranscriptional level, have fundamental roles in tumor progression [10, 11]. MiR-21 was described as an oncomiR and implicated in tumor progression of various cancers [12–15]. Of interest, up-regulation of miR-21 was associated with lung cancer diagnosis and prognosis, indicating a potential role of miR-21 in human lung cancer progression [16, 17]. Recent studies reported the involvement of miR-21 in LPS-induced immune response [18, 19]. These findings indicated a possible involvement of miR-21 in LPS-induced lung cancer progression.

In this study, we characterized the effect of LPS on outgrowth of primary human lung cancer cells and evaluated the potential role of miR-21 in this process. We demonstrated that miR-21 was essentially required for tumor growth of primary human lung cancer. TLR4 activation by LPS in primary human lung cancer cells resulted in increased ROS production, which in turn induced up-regulation of miR-21 and led to tumor outgrowth. Our findings could facilitate further understanding of lung cancer pathogenesis and be helpful for developing novel therapeutic strategies for lung cancer.

## RESULTS

### LPS stimulation promotes tumor outgrowth of primary human lung cancer

**Figure 1:**
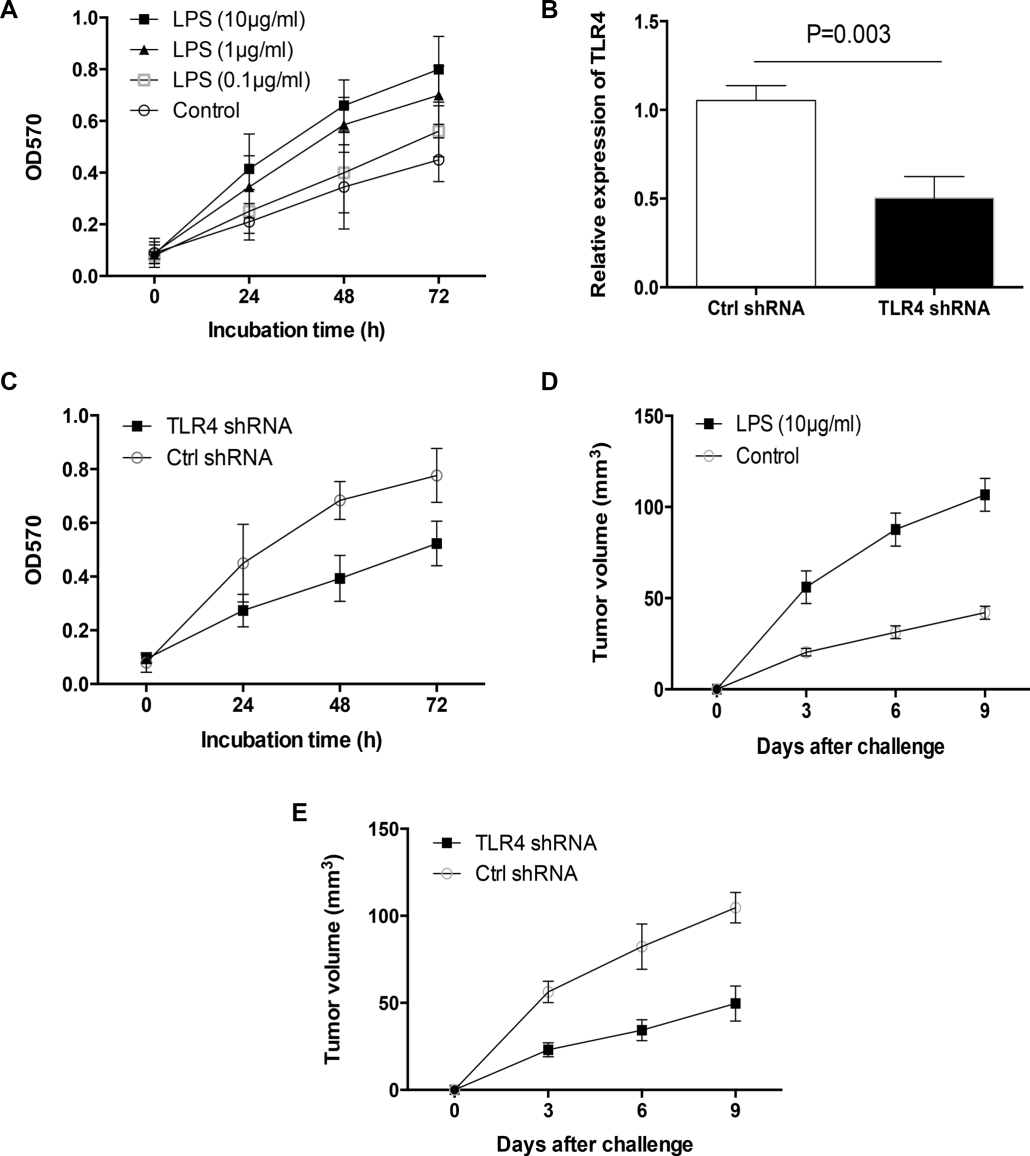
Activation of TLR4 with LPS promoted primary human lung cancer outgrowth (**A**) Freshly isolated human lung cancer cells from different tissues (*n* = 3) were treated with the indicated dose of LPS and detected for their outgrowth with MTT assay. (**B**) Human lung cancer cells freshly isolated from different tissues (*n* = 3) were transfected with TLR4 shRNA or control shRNA for 12 h, and detected for their TLR4 expression with qPCR. (**C**) Freshly isolated human lung cancer cells from different tissues (*n* = 3) were transfected with TLR4 shRNA or control shRNA for 24 h, stimulated with LPS (10 μg/ml), and detected for their growth with MTT assay. (**D**) Freshly isolated human lung cancer cells from different tissues (*n* = 6) were pretreated with or without LPS (10 μg/ml) for 24 h and injected into nude mice. Tumor volumes were determined at the indicated time. Data represented the mean (± SD) from 6 mice per group. (**E**) Primary human lung cancer cells freshly isolated from different tissues (*n* = 6) were transfected with TLR4 shRNA or the control, treated with LPS (10 μg/ml) for 24 h and then injected into nude mice. Tumor volumes at the indicated time were shown as mean (± SD) from 6 mice per group.

To explore the potential role of LPS in outgrowth of primary human lung cancer, freshly isolated human lung cancer cells were stimulated with an increasing dose of LPS and detected for their growth capacity. Results showed that LPS stimulation promoted tumor growth of primary human lung cancer cells in a dose dependent manner (Figure 1A). TLR4 was readily detectable in these primary human lung cancer cells and could be up-regulated by LPS stimulation (Supplementary Figure S1A). To detect the potential role of TLR4 signaling in LPS-mediated tumor outgrowth, primary human lung cancer cells were transfected with TLR4 shRNA or control and then stimulated with LPS. We found that transfection of TLR4 shRNA reduced TLR4 expression at both mRNA and protein levels (Figure 1B, Supplementary Figure S1B), and limited LPS-induced tumor outgrowth (Figure 1C). These data suggest TLR4 as the crucial receptor of LPS-induced primary human lung cancer outgrowth.

To confirm the effect of LPS on primary lung cancer outgrowth *in vivo*, freshly isolated human lung cancer cells were pretreated with or without LPS and injected subcutaneously into nude mice. We found that treatment with LPS promoted growth capacity of primary human lung cancer cells *in vivo* (Figure 1D). Knockdown of TLR4 blocked the enhanced tumor growth of primary human lung cancer *in vivo* (Figure 1E).

In essence, these results demonstrate an enhanced outgrowth of primary human lung cancer by LPS trigged TLR4 signaling.

### MiR-21 is required for LPS-mediated primary human lung cancer outgrowth

**Figure 2:**
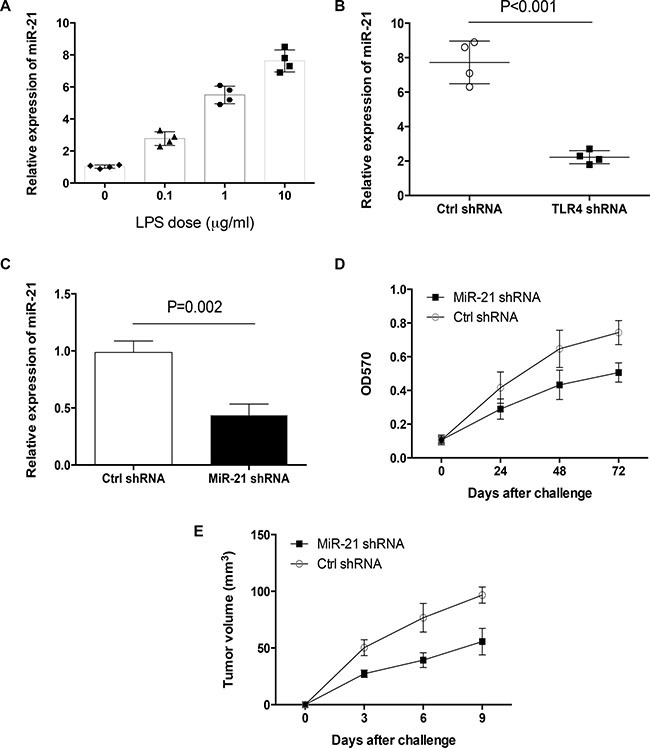
MiR-21 licensed LPS-mediated primary lung cancer outgrowth (**A**) Human lung cancer cells freshly isolated from different tissues (*n* = 4) were treated with the indicated dose of LPS for 24 h and detected for their expressions of miR-21 with qPCR. (**B**) Freshly isolated human lung cancer cells from different tissues (*n* = 4) were transfected with TLR4 shRNA or the control, stimulated with LPS (10 μg/ml) for 24 h and detected for miR-21 expressions. (**C**) Freshly isolated human lung cancer cells from different tissues (*n* = 3) were transfected with miR-21 shRNA or control shRNA for 24 h, and detected for miR-21 expressions. (**D**) Freshly isolated human lung cancer cells from different tissues (*n* = 3) were transfected with miR-21 shRNA or control shRNA, stimulated with LPS (10 μg/ml) and assayed for their growth capacity. (**E**) Freshly isolated human lung cancer cells from different tissues (*n* = 6) were transfected with miR-21 shRNA or control, treated with LPS (10 μg/ml) for 24 h and injected into nude mice. Tumor volumes at the indicated time were shown as mean (± SD) from 6 mice.

To evaluate the potential role of miR-21 in LPS-induced primary tumor outgrowth, we detected the expression of miR-21 in freshly isolated human lung cancer cells in response to LPS. We observed that LPS increased miR-21 expression in a dose dependent manner (Figure 2A). Knockdown of TLR4 abrogated the increased miR-21 expression by LPS in primary human lung cancer cells (Figure 2B). When primary human lung cancer cells were transfected with miR-21 shRNA and stimulated with LPS, we found that transfection with miR-21 shRNA reduced miR-21 expression and blocked LPS-induced tumor outgrowth (Figure 2C, 2D).

To confirm the potential role of miR-21 in LPS-induced primary human lung cancer outgrowth *in vivo*, nude mice were challenged with freshly isolated human lung cancer cells that were transfected with miR-21 shRNA and pretreated with LPS. Results showed that down-regulation of miR-21 attenuated LPS-enhanced tumor growth of primary human lung cancer in nude mice (Figure 2E). Collectively, these results reveal that increased miR-21 is required for LPS to enhance tumor growth of primary human lung cancer.

### MiR-21 licenses outgrowth of primary human lung cancer

**Figure 3:**
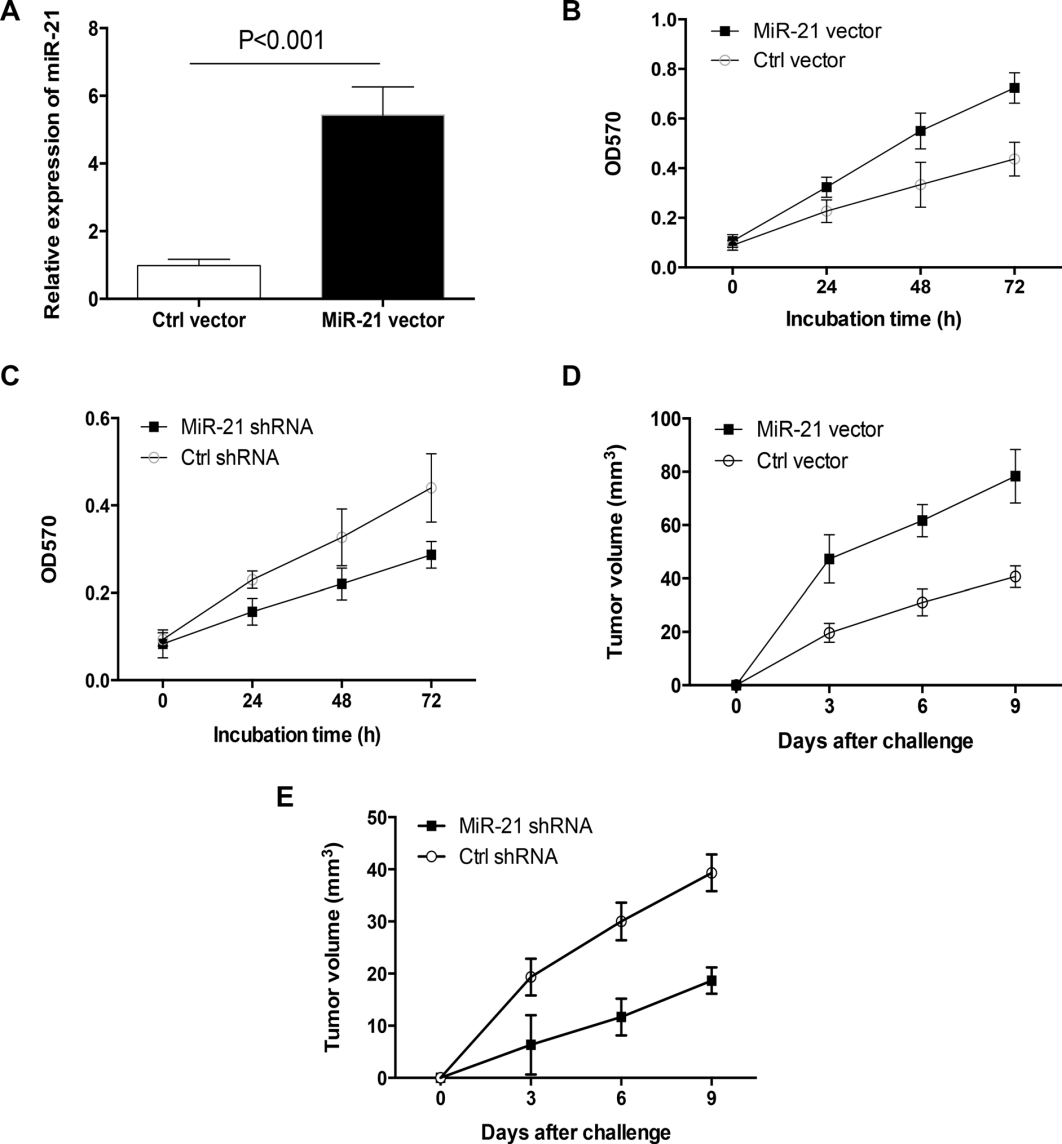
MiR-21 was crucial for primary lung cancer outgrowth (**A**) Freshly isolated human lung cancer cells from different tissues (*n* = 3) were transfected with miR-21 expression vector or the control for 24 h, and detected for their miR-21 expressions. (**B**) Freshly isolated human lung cancer cells from different tissues (*n* = 3) were transfected with miR-21 expression vector or control, and assayed for their outgrowth at the indicated time. (**C**) Freshly isolated human lung cancer cells from different tissues (*n* = 3) were transfected with miR-21 shRNA or the control and assayed for their outgrowth. (**D**, **E**) Freshly isolated human lung cancer cells from different tissues (*n* = 6) were transfected with miR-21 expression vector, miR-21 shRNA or the controls and injected into nude mice. Tumor volumes (mean ± SD) at the indicated time were determined from 6 mice per group.

Given the important role of miR-21 in LPS-induced tumor outgrowth, we detected the function of miR-21 itself in primary lung cancer growth. Transfection of freshly isolated human lung cancer cells with miR-21 expression vector resulted in elevated expression of miR-21 and enhanced tumor outgrowth (Figure 3A, 3B). Transfection with miR-21 shRNA ameliorated outgrowth of primary human lung cancer (Figure 3C). To confirm these results *in vivo*, nude mice were injected with primary human lung cancer cells that were transfected with miR-21 expression vector or miR-21 shRNA respectively. We found that increased miR-21 expression enhanced tumor outgrowth, while decreased miR-21 expression attenuated tumor outgrowth *in vivo* (Figure 3D, 3E). These findings regard miR-21 as an essential oncomiRNA for tumor growth of primary human lung cancer.

Phosphatase and tensin homolog (PTEN) and programmed cell death 4 (PDCD4) are well-known targets of miR-21 [20, 21]. We determined whether PTEN and PDCD4 were critical targets of miR-21 in primary human lung cancer cells. Results showed that transfection with miR-21 expression vector decreased expressions of PTEN and PDCD4 (Supplementary Figure S2A, S2B). Vice versa, transfection with miR-21 shRNA increased expressions of PTEN and PDCD4 (Supplementary Figure S2C, S2D). Of important, overexpression of PTEN and PDCD4 blocked the pro-cancer activity of miR-21 in lung cancer outgrowth (Supplementary Figure S2E). These data identify PTEN and PDCD4 as important targets of miR-21 in primary human lung cancer.

### Elevated ROS production confers enforced miR-21 expression by LPS

**Figure 4:**
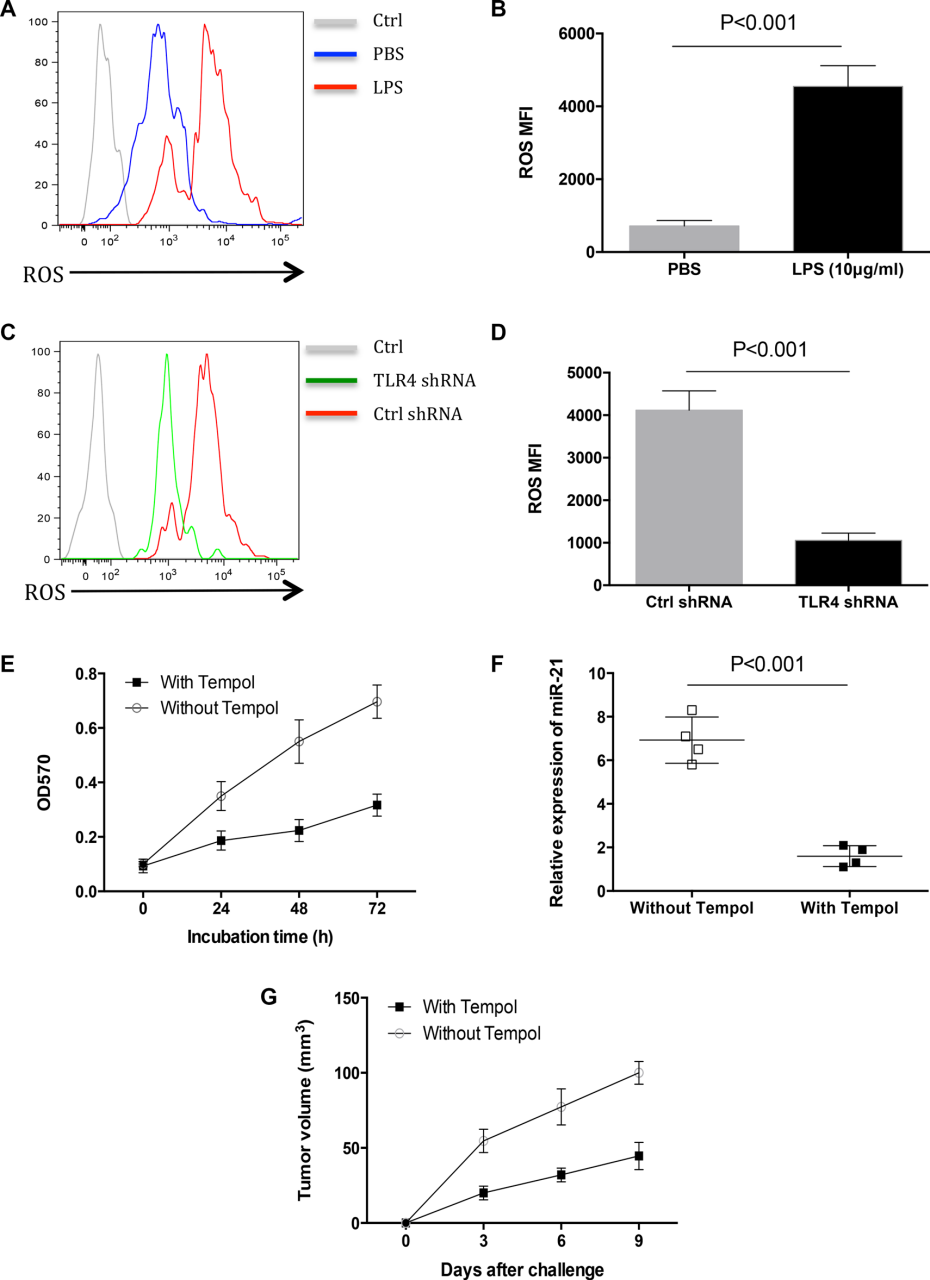
ROS conferred increased miR-21 expression by LPS stimulation (**A**, **B**) Human lung cancer cells freshly isolated from different tissues (*n* = 3) were treated with or without LPS (10 μg/ml) for 24 h and detected for their ROS levels with flow cytometry. (**C**, **D**) Human lung cancer cells freshly isolated from different tissues (*n* = 3) were transfected with TLR4 shRNA or control shRNA, stimulated with LPS (10 μg/ml) for 24 h and detected for ROS generation. (**E**) Human lung cancer cells freshly isolated from different tissues (*n* = 3) were treated with LPS (10 μg/ml) in the presence or absence of Tempol (50 μM) for 24 h, and detected for their growth with MTT assay. (**F**) Human lung cancer cells freshly isolated from different tissues (*n* = 3) were treated with LPS (10 μg/ml) in the presence or absence of Tempol (50 μM) for 24 h, and assayed for their miR-21 expressions. (**G**) Human lung cancer cells freshly isolated from different tissues (*n* = 6) were treated with LPS (10 μg/ml) in the presence or absence of Tempol (50 μM) for 24 h, and injected into nude mice. Tumor volumes (mean ± SD) were determined from 6 mice per group.

Reactive oxygen species (ROS) were involved in TLR4-triggered immune response and gastric cancer progression [22, 23]. We detected the ROS levels in freshly isolated human lung cancer cells in response to LPS. Results showed that LPS induced ROS production in primary human lung cancer cells (Figure 4A, 4B). Knockdown of TLR4 abrogated the LPS-increased ROS production (Figure 4C, 4D). To evaluate the function of ROS in LPS-induced primary human lung cancer outgrowth, freshly isolated human lung cancer cells were treated with LPS in the presence of Tempol as a ROS scavenger. Results showed that Tempol abrogated LPS-enhanced primary lung cancer outgrowth (Figure 4E). We detected whether ROS was required for LPS-induced miR-21 expression, and found that Tempol inhibited the elevated miR-21 expression in response to LPS (Figure 4F).

To determine the effect of ROS on LPS-induced primary human lung cancer outgrowth *in vivo*, nude mice were injected with freshly isolated human lung cancer cells that were pretreated with LPS in the presence or absence of Tempol. Results showed that Tempol abrogated the enhanced tumor outgrowth induced by LPS *in vivo* (Figure 4G).

Collectively, elevated ROS production is critical for LPS to induce miR-21 expression and primary lung cancer outgrowth.

### TLR4 expression is correlated with miR-21 expression and ROS production in lung cancer patients

**Figure 5:**
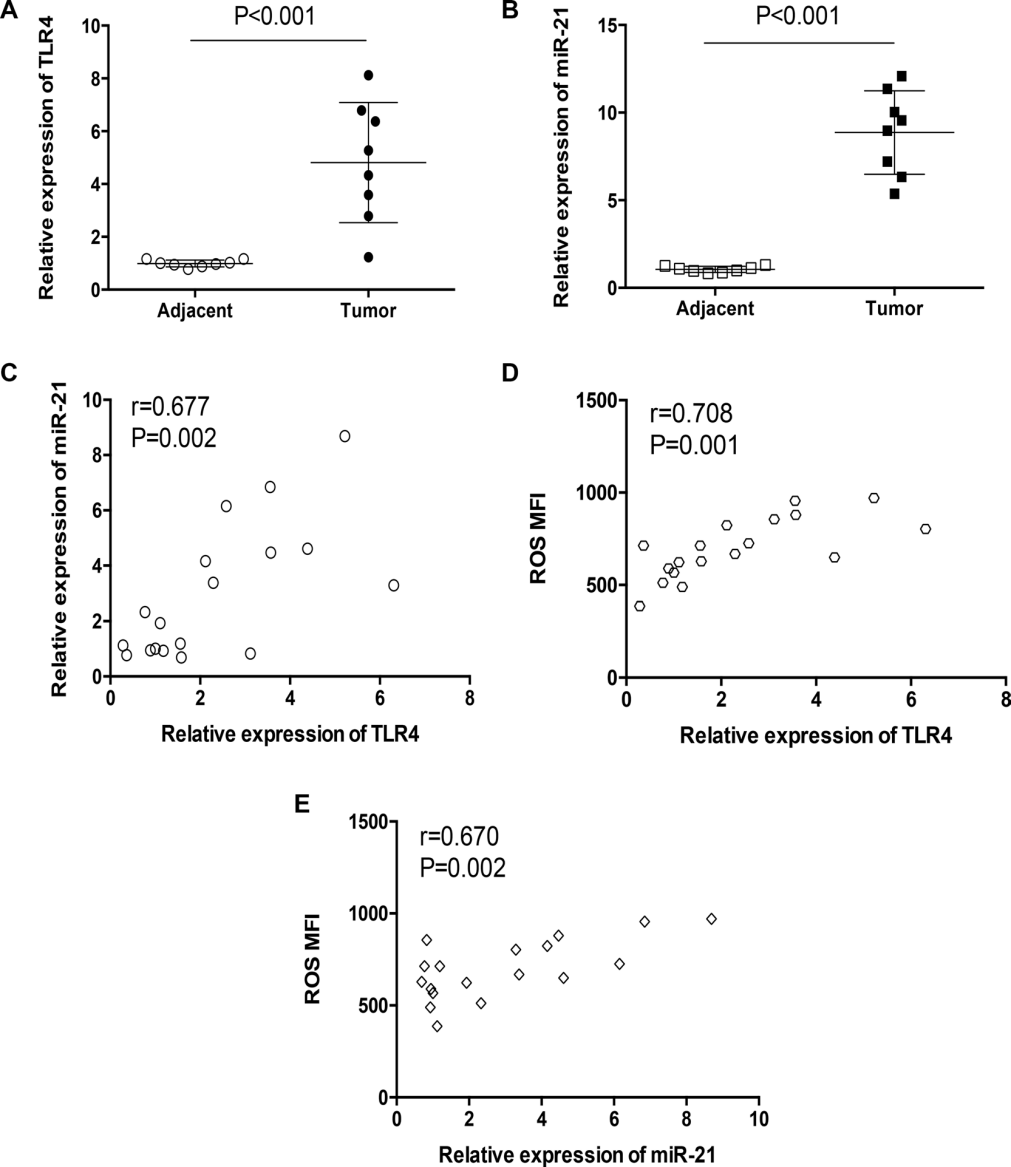
TLR4 expressions correlated with miR-21 expression and ROS production in clinical patients (**A**, **B**) The expressions of TLR4 and miR-21 in tumor tissues and adjacent tissues were determined in 8 clinical patients with qPCR. (**C**–**E**) The correlations between TLR4 expression levels and miR-21 expression levels, as well as ROS production, in freshly isolated, untreated human lung cancer cells were analyzed in 18 clinical patients. One dote represents the data from one patient.

To elucidate the *in vivo* relevance of our findings in clinical patients, we detected expressions of TLR4 and miR-21 in tumor tissues and adjacent tissues. Results showed that TLR4 and miR-21 expressions were significantly higher in tumor tissues (Figure 5A, 5B). Further, freshly isolated, untreated human lung cancer cells were assayed for expressions of TLR4, miR-21 and ROS levels. Analysis showed that TLR4 expression levels were correlated with their miR-21 expressions and ROS levels (Figure 5C, 5D). ROS levels in primary human lung cancer cells were correlated with their miR-21 expressions (Figure 5E). These results suggest an involvement of TLR4/ROS/miR-21 pathway in tumor progression of lung cancer patients.

## DISCUSSION

Herein, we demonstrated that LPS promoted tumor outgrowth of primary human lung cancer though TLR4 signaling. Activation of TLR4 with LPS resulted in elevated ROS production in primary human lung cancer cells, which increased miR-21 expression and tumor outgrowth. The close correlation of TLR4 expression with miR-21 and ROS levels in freshly isolated, untreated human lung cancer cells suggested a TLR4/ROS/miR-21 pathway through which Gram-negative bacteria facilitates lung cancer progression in clinical patients.

Lung cancer patients are frequently present with pulmonary infection of Gram-negative bacteria, which predicts poor prognosis [24]. Mechanistically, a growing body of literature suggested that pulmonary inflammation could contribute to lung cancer progression [4, 25–27]. In current study, our results derived from freshly isolated human lung cancer cells demonstrated the direct pro-cancer effect of LPS on tumor outgrowth. These findings closely reflected the pro-cancer function of Gram-negative bacteria in lung cancer progression. In support, heat-inactivated E. coli increased growth capacity of primary human lung cancer cells (Supplementary Figure S3). Further, we identified miR-21 as an effective regulator of primary human lung cancer outgrowth. Although we did not exclude other possible factors, miR-21 was required for LPS-induced lung cancer outgrowth. In consistent, elevated miR-21 expression was a biomarker with diagnostic and prognostic value for many cancers including lung cancer [12–15]. MiR-21 increased proliferation, invasion and migration of lung cancer [28–30]. Combing these studies suggest miR-21 as a promising target for developing lung cancer therapeutics.

ROS, which are dominantly produced by mitochondria and Consistently, NADPH oxidase, are widely considered cytotoxic and in high levels they induce cell damage [31, 32]. However, studies within cancer revealed that higher levels of ROS were involved in tumor pathogenesis [33–35]. In this study, we extended previous studies by demonstrating that elevated ROS production in freshly isolated human lung cancer cells was essential for LPS to promote tumor outgrowth. Increased ROS generation relied on NADPH oxidase activity as NADPH oxidase inhibitor DPI, but not mitochondrial complex I inhibitor Rotenone, blocked LPS-induced ROS generation (Supplementary Figure S4). NADPH oxidase 1-dependent ROS was crucial for TLR4 signaling triggered tumor metastasis of human lung cancer [5]. Combing these results suggested that TLR4 activation in response to LPS led to enforced NADPH oxidase 1 activity, which in turn increased ROS production and human lung cancer progression. Targeting ROS was a potential strategy to control tumor progression, especially for lung cancer patients with Gram-negative bacteria inflammation. In support, NADPH oxidase inhibitor DPI effectively abrogated tumor metastasis of human lung cancer cells [36]. Besides, we determined that elevated ROS production in primary human lung cancer cells conferred increased expression of miR-21 in response to LPS. In line with our findings, miR-21 was reported as an important target of ROS, which contributed to the highly invasive and metastatic phenotype of cancer cells [37, 38]. Germane to the underlying mechanisms, NADPH oxidase-dependent ROS generation led to increased miR-21 expression through AKT pathway in tumor cells [37]. ROS generation could induce activation of ERK/NF-kB pathway, which, in turn, increased miR-21 expression [39]. ROS-mediated ERK and p38 phosphorylation was critical for UVB-induced miR-21-PDCD4 signaling [40]. Thus, ROS production in response to LPS might up-regulate miR-21 expression through AKT, NF-kB and MAPK pathways in primary human lung cancer cells. However, miRNA-21 was also capable of modulating ROS generation by targeting SOD3 and TNFα [41], indicating a complex “cross-talk” between ROS generation and miR-21 expression.

We should acknowledge some limitations of this study. Current findings with freshly isolated human lung cancer cells explored short-term pro-cancer function of LPS in tumor outgrowth. The continuous and long-time lasting effects are unknown. Precise mechanisms for enforced ROS generation by LPS and subsequent increased miR-21 expression in primary human lung cancer cells still remain unclear. In addition, considering the diversity and complexity of clinical patients, the sample size is relatively low and further study with large number of clinical samples would substantiate these findings.

In summary, our current findings are learned from primary lung cancer cells freshly isolated from surgical tissues but not lung cancer cell lines, and thus closely reflect clinical relevance in patients. We demonstrate an essential role of TLR4/ROS/miR-21 pathway in LPS-induced primary human lung cancer outgrowth. These results reveal a new mechanism through which Gram-negative bacteria facilitate lung cancer pathogenesis and provide clues for developing new therapeutics for lung cancer patients.

## MATERIALS AND METHODS

### Ethics statement

All patients provided their written informed consent to participate in this study. Studies with clinical samples were performed in accordance with the ethical standards laid down in the 1964 Declaration of Helsinki and its later amendments. Animal experiments were performed according to the Guide for Care and Use of Medical Laboratory Animals. Human and animal experiments were performed with an approval from Tongji Institutional Ethics Committee.

### Patients

Totally 50 human lung cancer patients with pulmonary infection of Gam-negative bacteria were enrolled in this study. The diagnosis of lung cancer was confirmed by pathology. Clinical information of lung cancer patients was summarized in Table 1. Subjects with autoimmune diseases or under immunosuppressive treatment were excluded.

**Table 1:** Clinical characteristics of lung cancer patients

Clinical parameters	Number
Sex	
Male	35
Female	15
Age (years)	49–73
Median	62.8
Stages	
I	3
II	36
III	9
IV	2
Histological type	
Adenocarcinoma	37
Others	13

1 Clinical stage is according to TNM stage.

### Mice

Female BALB/c nude mice of 6–8 weeks old were purchased from Shanghai Laboratory Animal Center, CAS. All mice were housed under specific pathogen-free conditions.

### Cell culture and reagents

Human lung cancer cells were freshly isolated from surgical tissues with Clonogenic Tumor Cell Isolation Kit (Cell Biolabs) according to the manual's instructions. Cells were cultured in complete RPMI 1640 medium containing 10% heat-inactivated fetal bovine serum (Gibco) supplemented with 2 mM glutamine, 100 IU/ml penicillin and 100 mg/ml streptomycin sulfate at 37°C under 5% CO2. Lipopolysaccharide (LPS) was purchased from Sigma. psiRNA vector expressing shRNA targeting human TLR4 gene (psirna42-htlr4) and control were purchased from Invivogen. Expression vector for human miR-21 (MI0000077) and control were purchased from Origene. Human miR-21 shRNA (shADV-215486) and control were purchased from Vector Biolabs. Tempol was purchased from Santa Cruz. All reagents were used according to manual's instructions.

### Tumor outgrowth *in vitro*

Tumor outgrowth *in vitro* was detected by analyzing proliferative expansion of primary human lung cancer cells with MTT assay. Briefly, cells were seeded at 4 × 10^3^ cells each well and incubated in the presence or absence of LPS (0.1–10 μg/ml) in 96-well plates for 72 h. Assessment of cell proliferation was measured with MTT cell proliferation kit (Cayman Chemical) according to the manual's instructions.

### Tumor outgrowth *in vivo*

Tumor outgrowth of primary human lung cancer cells in nude mice was assessed as previously described [42]. Briefly, groups of nude mice were subcutaneously injected with 4 × 10^6^ primary human lung cancer cells suspended in 200 μl of PBS. Control mice receive equal volume of PBS. Immediately before subcutaneous injection, primary human lung cancer cells were treated with LPS (10 μg/ml) for 24 h or PBS as a control. The size of the tumor was measured at days 3, 6 and 9 after implantation by digital calipers. Each tumor measurement was taken in duplicate by two different investigators.

### Real-time PCR

Total RNA was extracted with RNeasy Mini Kit (Qiagen). cDNA was synthesized with Maxima First Strand cDNA Synthesis Kits (Thermo Scientific). The primers were obtained from Applied Biosystems. Quantitative PCR analyses were carried out in duplicate to detect mRNA expression with Maxima SYBR Green qPCR Master Mixes (Thermo Scientific). GAPDH was used as an internal control. TaqMan miRNA assays (Applied Biosystems) were used to detect miRNA expression levels with U6 RNA as an internal control.

### Flow cytometry

For flow analysis, 0.5 million cells were stained with PE-conjugated anti-human TLR4 antibody (12-9917-42, eBioscience) or the isotye control (12-4724-81, eBioscience), and analyzed on a FACSCalibur flow cytometer (BD). ROS levels were detected with CellROX^®^ Deep Red Reagent (Life Technologies) according to manual's instructions. Collected data were analyzed with FlowJo software (Tree Star).

### Statistical analyses

Data were presented as mean ± SD. Unpaired 2-tailed Student's *t*-test and Pearson correlation were used for statistical analyses using the program PRISM 6.0 (GraphPad Software Inc., San Diego, CA, USA). A value of *P* < 0.05 was considered statistically significant.

## SUPPLEMENTARY FIGURES


