# Supplementary material for: TLR4/ROS/miRNA-21 pathway underlies lipopolysaccharide instructed primary tumor outgrowth in lung cancer patients
Source: Oncotarget. 2016 Jun 7;7(27):42172–82. doi: 10.18632/oncotarget.9902 (PMC5173125; doi:10.18632/oncotarget.9902)
Supplement: Supplementary file 1 [file oncotarget-07-42172-s001.pdf]

TLR4/ROS/miRNA-21 pathway underlies lipopolysaccharide instructed primary tumor outgrowth in lung cancer patients

Supplementary Materials

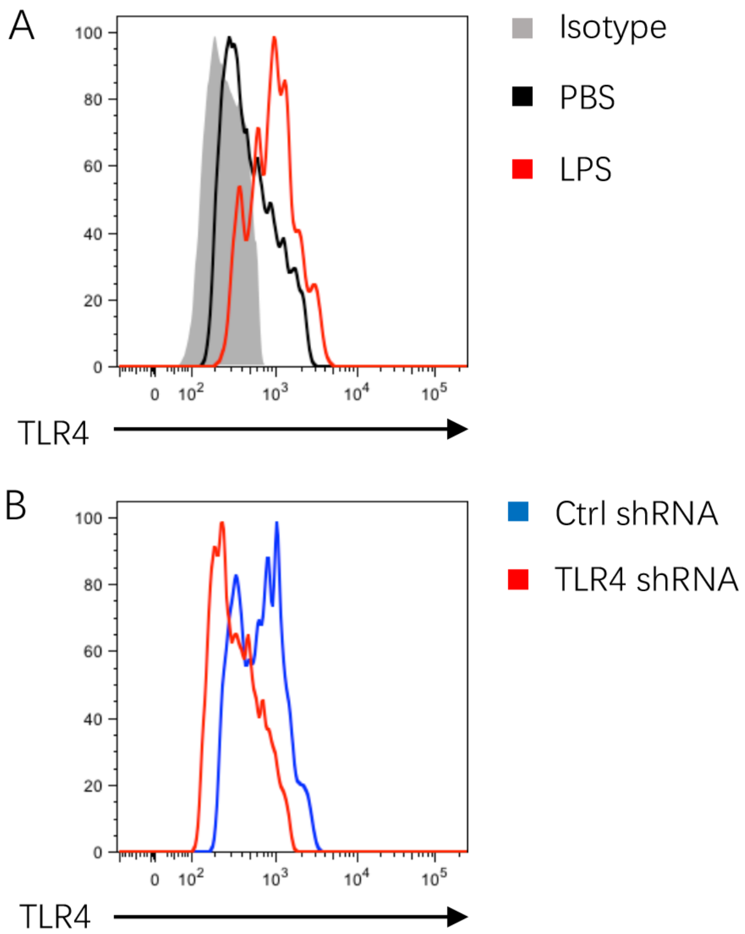

Supplementary Figure S1: TLR4 protein expression in human lung cancer cells. (**A**) Freshly isolated human lung cancer cells were treated with or without LPS (10 μg/ml) for 24 h and detected for TLR4 expression by flow cytometry. (**B**) Freshly isolated human lung cancer cells were transfected with TLR4 shRNA or control shRNA for 24h and analyzed for TLR4 protein expressions.

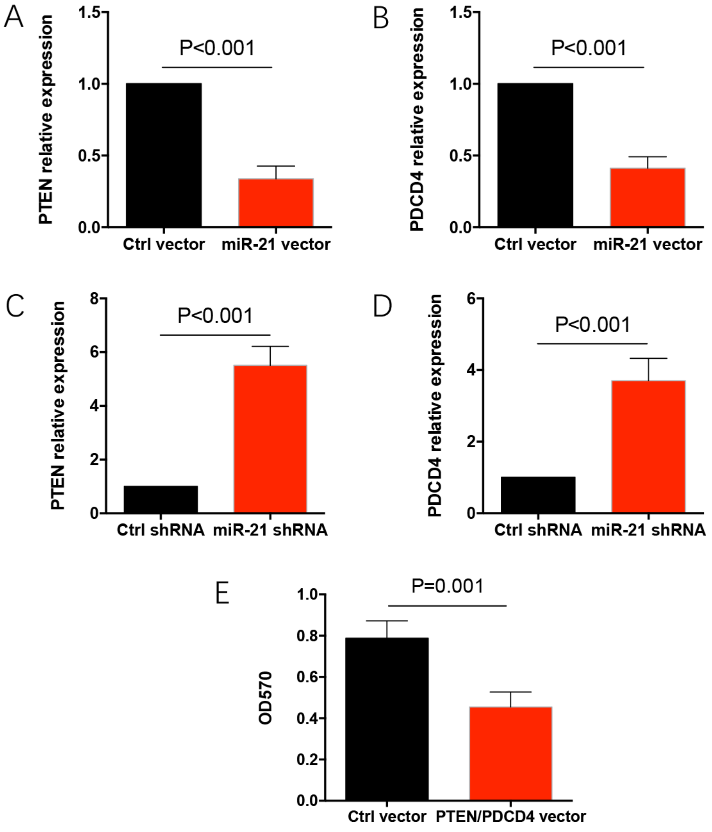

Supplementary Figure S2: PTEN and PDCD4 were critical targets of miR-21 in human lung cancer. (**A–D**) Freshly isolated human lung cancer cells from different surgical tissues (*n* = 4) were transfected with miR-21 expression vector, miR-21 shRNA or controls for 12 h, and detected for their expressions of PTEN and PDCD4. (**E**) Freshly isolated human lung cancer cells from different surgical tissues (*n* = 4) were transfected with miR-21 expression vector plus PTEN and PDCD4 expression vectors (Origene), and detected for their growth capacity for 72 h.

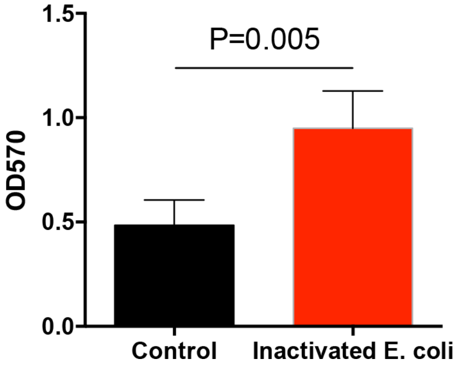

Supplementary Figure S3: Gram-negative bacteria promoted outgrowth of human lung cancer. Freshly isolated human lung cancer cells from different surgical tissues (***n*** = 4) were treated with or without heat-inactivated E. coli (10^8 CFU/ml) for 72 h, and detected for their growth capacity. Collective data were shown as mean ± SD.

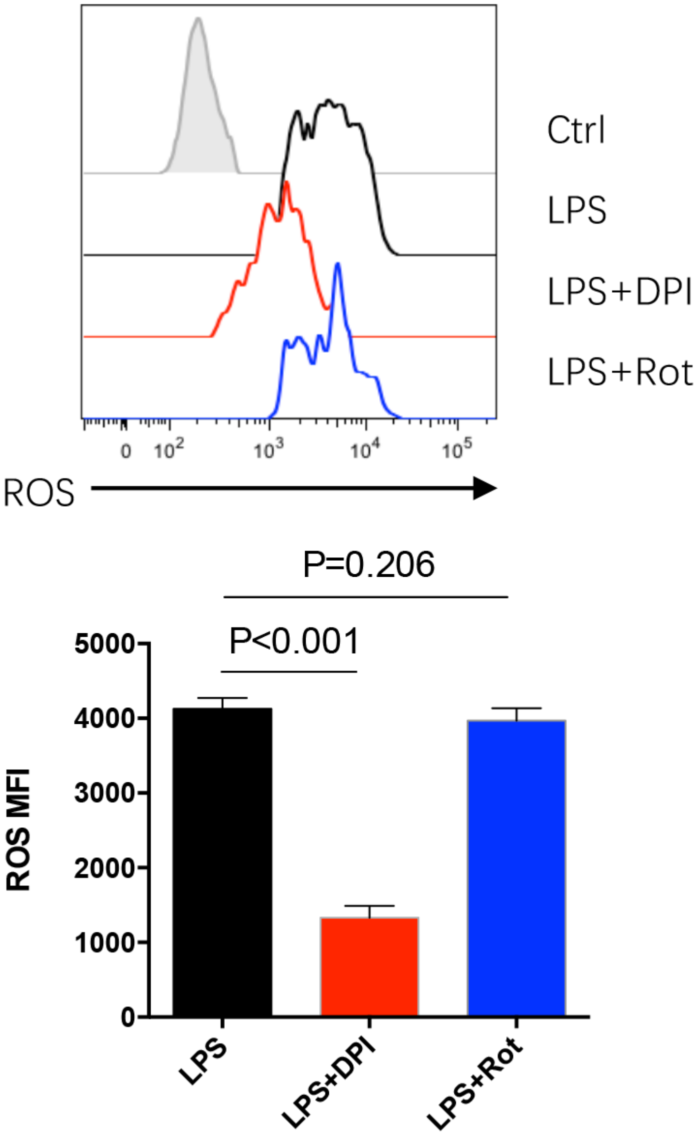

Supplementary Figure S4: NADPH oxidase conferred ROS generation in response to LPS. Freshly isolated human lung cancer cells from different surgical tissues (*n* = 4) were treated with LPS (10 µg/ml) in the presence of DPI (10 µM, Sigma) or Rotenone (2 µM, Sigma) for 24 h, and detected for their ROS production. Representative image and collective data (mean ± SD) were shown.
